# Utilizing cohort-level and individual networks to predict best response in patients with metastatic triple negative breast cancer

**DOI:** 10.1038/s41698-025-00959-w

**Published:** 2025-06-13

**Authors:** Daniel Bottomly, Christina Zheng, Allison L. Creason, Zahi I. Mitri, Gordon B. Mills, Shannon K. McWeeney

**Affiliations:** 1 Knight Cancer Institute, Oregon Health & Science University, Portland, OR USA; 2 Department of Biomedical Engineering, Oregon Health & Science University, Portland, OR USA; 3 Division of Medical Oncology, University of British Columbia, Vancouver, BC Canada; 4 British Columbia Cancer Agency, Vancouver, BC Canada; 5 Department of Cell, Developmental & Cancer Biology, Oregon Health & Science University, Portland, OR USA; 6 Division of Oncological Sciences, Oregon Health & Science University, Portland, OR USA

**Keywords:** Computational biology and bioinformatics, Oncology

## Abstract

Given the highly aggressive and heterogeneous nature of metastatic triple-negative breast cancer, molecular subtypes have been evaluated for their utility in patient stratification and therapeutic selection. Leveraging both our unique longitudinal multimodal analysis of serial tumor biopsies, as well as existing public reference cohorts, we refined clinically relevant molecular subtypes through de-novo network-based approaches. A plasma/B-cell related co-expression module emerged as a robust predictor of clinical response. Refinements of this module were significantly associated with pathological complete response and survival in the CALGB and METABRIC cohorts, as well as dramatically improving the call rate in a CLIA setting. We explored patient-specific networks to monitor individual adaptive responses to therapy, allowing for dynamic adjustments in treatment strategies. Our work supports the shift from traditional molecular subtyping towards a more integrated view that includes the tumor microenvironment and immune landscape in a network-based context.

## Introduction

Triple negative breast cancer (TNBC), characterized by a lack of expression of estrogen (ER) and progesterone (PR) receptors and a lack of human epidermal growth factor receptor 2 (HER2) amplification, is the most aggressive breast cancer subtype with the least favorable outcomes. TNBC is also characterized by high tumor heterogeneity, which has made the development of therapies that provide a durable response challenging. The development of a TNBC molecular classification system for patient stratification has been an area of focus over the last two decades^1–5^.

Our phase II clinical trial (NCT03801369; Adaptive multi-drug treatment of evolving cancers (AMTEC)) is evaluating the efficacy of the combination of the Poly (ADP-ribose) polymerase (PARP) inhibitors (PARPi) olaparib and the programmed death-ligand 1 (PD-L1) inhibitor durvalumab for the treatment of BRCA^wt^ metastatic TNBC (mTNBC) patients with a longitudinal analysis of serial tumor samples in real-time to identify adaptive mechanisms of resistance as they emerge in response to treatment. This longitudinal characterization includes comprehensive multimodal analysis of serial liquid and tumor biopsies utilizing the Oregon Health & Science University Knight Cancer Institute precision oncology platform, Serial Measurements of Molecular and Architectural Responses to Therapy (SMMART).

Initial analysis of the AMTEC data^6^ indicated that one of the most informative predictors of response was the molecular subtypes Basal-Like Immune Activated (BLIA), Luminal Androgen Receptor (LAR) or Basal-Like Immunosuppressed (BLIS), termed the Burstein subtypes^4^. The Burstein expression subtypes were originally identified in TNBC tumors, with those with the BLIS or LAR subtypes having poor prognosis while BLIA tumors had improved outcomes^4^. In AMTEC, we used collapsed versions of these subtypes, which were seen to correspond to poor survival outcomes (BLIS/LAR) vs better survival outcomes (Non-BLIS/LAR). Although they were highly predictive in our cohort, one challenge with the Burstein subtypes in the clinical setting (i.e., CLIA laboratory) was the classification of a subset of patient samples correlated with multiple subtypes. Currently, they are given a “No Call” or “Indeterminant (IND)” in the CLIA setting by our diagnostic laboratory and not reported (12/26 (46.2%) patient samples) for clinical use.

We wanted to determine if the BLIA/BLIS/LAR subtypes could be further refined and reduce the number of “No Call” determinations. In order to explore the prognostic immune signatures displayed by our multi-modal dataset, we examined the utility of de-novo coexpression network-based approaches given that progression would likely reflect underlying perturbations of complex intracellular networks. Recognizing the limited sample size in the AMTEC trial for network inference, we leveraged The Cancer Genome Atlas (TCGA) Breast Cancer samples^7^ to create a reference (pre-treatment) cohort. We evaluated the network-based signatures as predictors of best clinical response. In addition, we wanted to assess the utility of patient specific networks to allow us to identify individual network rewiring (due to adaptive responses to perturbation such as treatment) which could be utilized for monitoring disease outcome and therapy selection in our precision oncology tumor boards.

## Results

### Development of a reference cohort and reference networks

**Fig. 1:**
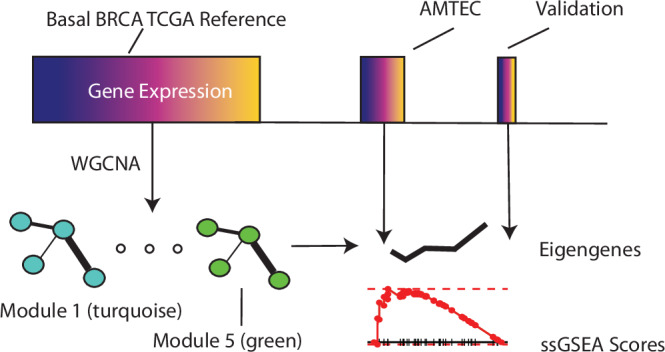
Overall workflow for co-expression module formation. A Basal-classified BRCA TCGA reference cohort was selected (*n* = 152) and used to form WGCNA co-expression subnetworks (modules) labeled in terms of numbers 1–5 and colors. These subnetworks enabled the scoring of the AMTEC cohort in terms of expression summaries (eigengenes; PC1) and single-sample gene set enrichment. The subnetworks vary in size: Mod1 (turquoise) has 469 genes, Mod2 (blue) has 323, and Mod3 (brown) has 190 genes while Mod4 (yellow) and Mod5 (green) have only 48 and 42, respectively.

We leveraged 152 Basal-classified Cancer Genome Atlas (TCGA) Project Breast Cancer samples^7^ to provide a pre-therapy reference cohort for assessment of the degree of therapeutic changes in clinical trial patient samples. Given that these therapy-related expression perturbations often lead to the rewiring or alteration of relevant gene networks, we performed weighted gene co-expression network analysis (WGCNA^8^) to provide the pre-treatment reference networks and identified five topologically significant gene expression modules (also known as subnetworks) across the basal TCGA samples (See Fig. 1 for a diagram of the design and Supplementary Data S1 for gene module membership) and **Methods** for description of topological significance.

### Clinical trial cohort

We leveraged 13 patients with metastatic triple negative breast cancer (mTNBC) from the phase II clinical trial (NCT03801369; Adaptive multi-drug treatment of evolving cancers (AMTEC))^6,9,10^ each having paired pre-treatment (Bx1) and on-treatment (combination of olaparib and durvalumab; Bx2) samples (denoted as the “AMTEC cohort”). Additionally, we formed a separate group of 10 patient samples (5 Bx1 and 5 Bx2, 3 of which were paired) termed the “Validation cohort”. For this study, the main outcome was based on the best response achieved by a given patient as part of the trial. These outcomes were defined as either progressive disease (PD), stable disease (SD), or partial response (PR). For classification, we further binned patients into those who achieved a best response of SD or PR, termed responders, vs those who did not (PD), termed non-responders.

### Module characterization

**Fig. 2:**
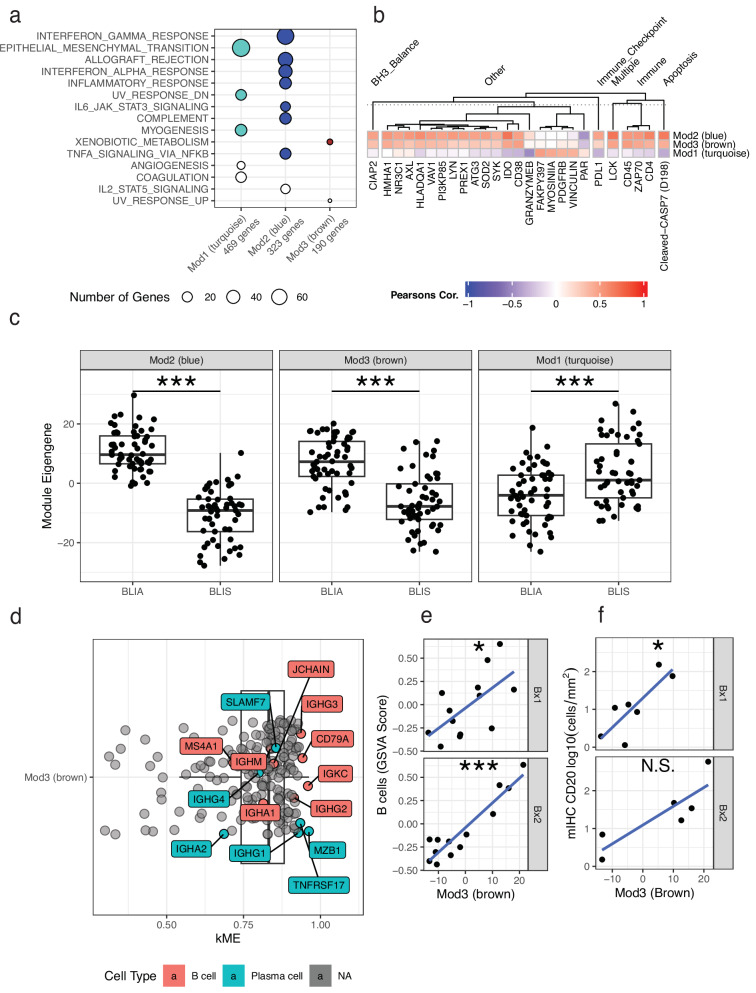
Co-expression modules formed from Basal classified TCGA reflect EMT and Immune processes. **a** Modules (X-axis) with an unadjusted enrichment *p*-value < 0.05 with respect to any MSigDB Hallmark (Y-axis) are shown. Dots are sized according to the number of overlapping genes between the module and Hallmark. Colors indicate significant enrichment (FDR < 0.05). **b** The correlation between module eigengenes (rows) and RPPA antibodies (columns) is displayed and clustered within pathway requiring at least one entry to have a Pearson’s correlation > 0.4. **c** The module eigengenes are significantly different (Welch’s T-test; all *P*-values < 0.001; *n* = 110) between the called Burstein BLIA and BLIS subtypes in Basal-classified TCGA. Mod3 (Brown) is an immune-related module associated with a B/plasma-cell signature. **d** Several B/plasma-cell markers specific are amongst the most influential genes as indicated by their correlation with the module eigengene (kME). **e** A significant Pearson’s correlation is seen for both Bx1 (r = 0.622; *P*-value = 0.023; *n* = 13) and Bx2 (r = 0.933; *P*-value < 0.001; *n* = 13) when Mod3 (brown) is compared to a GSVA-scored list of markers for B cells in AMTEC. **f** The Mod3 (brown) eigengene is significantly correlated with CD20+ cell density from mIHC for Bx1 patient biopsies in AMTEC (Pearson’s correlation; r = 0.757; *P*-value = 0.049; *n* = 7). The symbols ‘*’, ‘**’ and ‘***’ indicates unadjusted *P*-values < 0.05, 0.01, and 0.001, respectively. ‘N.S.’ indicates not meeting significance criteria (*P*-value < 0.05).

Three of the five modules (subnetworks) were significantly enriched for MSigDB Hallmarks^11^ gene signatures (FDR < 0.05; Fig. 2a), therefore, for the purposes of this manuscript, those are the ones that were focused on. As the modules by definition are highly correlated gene sets, we computed the first two principal component (PC) scores for our three modules of interest and predicted the corresponding values for our AMTEC cohort to ensure compatibility. Both biopsies from AMTEC patients completely overlapped within the range of PC1 and PC2 values of TCGA (Supplementary Fig. S1). For both TCGA and AMTEC patients, we used the PC1 score as the representative value for each module, termed the “eigengene”^12^ or “module eigengene”.

Module 1 (Mod1 or Turquoise) was most significantly enriched for epithelial-to-mesenchymal transition (EMT). Module 2 (Mod2 or Blue) was enriched for allograft rejection, complement, inflammatory response, interferon alpha and gamma response, as well as TNFA and IL6-JAK-STAT3 signaling. While Module 3 (Mod3 or Brown) was only enriched for Xenobiotic metabolism. Correlating the modules with Reverse Phase Protein Array (RPPA) antibodies in TCGA showed that both Mod2 (Blue) and Mod3 (Brown) were associated with immune response while Mod1 (Turquoise) was characterized by genes influencing EMT processes such as Vinculin^13,14^ and PDGFR-B^15,16^ as well as cell adhesion such as MYOSIN1A^17^ and FAK^18^ (Fig. 2b). Modules 1-3 were likewise significantly associated with the “Burstein” BLIA and BLIS subtypes^4^ with Mod2 (Blue) and Mod3 (Brown) increased in BLIA relative to BLIS while Mod1 (Turquoise) was increased in BLIS (Fig. 2c). Interestingly, the Mod2 (Blue) eigengene by itself could predict BLIA/BLIS status in TCGA with 93.6% accuracy (MCC: 0.873, F1: 0.938) while Mod3 (Brown) was less predictive at 77.3% (MCC: 0.545, F1: 0.783) using single-feature logistic regression models. Overall, this indicated that although Modules 2 and 3 were related, Mod2 (Blue) serves as an immunomodulatory feature. The correlation of a gene’s expression profile with its assigned module eigengene (termed kME) can serve as a measure of membership in that module^12^. In practice, this means that high kME values indicate that the gene’s expression pattern closely mirrors its corresponding module eigengene. Given the uninformative of the MSigDB Hallmarks, we further assessed Mod3 (Brown) by annotating the most influential genes in the signature as indicated by their correlation with the module eigengene. As can be seen in Fig. 2d, the Mod3 (Brown) expression pattern reflects genes associated with B and Plasma cells, such as MZB1, CD79A, and MS4A1 (CD20), a standard B-cell marker. Significant Pearson’s correlations in both Bx1 (*P*-value = 0.023) and Bx2 (*P*-value < 0.001) were seen between the Mod3 (Brown) eigengenes and GSVA scores of a previously reported B-cell gene set^19^ (Fig. 2e). This was further confirmed by our multiplex immunohistochemistry (mIHC) data as the Mod3 (Brown) eigengene highly correlates with CD20+ cell density in AMTEC Bx1 samples (Fig. 2f). In total, this led to us to attributing Mod2 (Blue) and Mod3 (Brown) to distinct immune processes/cell types while Mod1 (Turquoise) likely represented EMT/cell adhesion processes.

Shepard et al.^20^ examined RNA sequencing from pre-treatment TNBC tumor biopsies as part of the CALGB 40603 clinical trial. After analyzing the B and T cell repertoire, they found that low diversity (in terms of the Evenness measure^20^—see below) of immunoglobulin G (IgG) was associated with both pathologic complete response and event-free survival^20^. To explore this further, we additionally analyzed the B and T cell repertoires in the AMTEC samples. In agreement with our characterization of Mod3 (Brown), the eigengene was significantly correlated (all *P*-values < 0.001) with the abundance of all three immunoglobulin chains (IGH, IGK, and IGL; Supplementary Fig. S2a). We also assessed diversity measures for the IgG class for AMTEC. Depending on the measure, patients whose best response was PD tended to have low abundance and trended towards lower or no difference in diversity as compared to responders (SD/PR) (Supplementary Fig. S2b).

The Evenness measure^20^, which is defined as the entropy normalized by the log of the number clonotypes, showed no difference between response groups (Supplementary Fig. S2c). Adjusting for the large difference in read counts for the IGH chain between samples by down-sampling, we observed that non-responders (PD) had marginally less (*P*-value = 0.048) Evenness in Bx1 samples compared to responders (SD/PR) (Supplementary Fig. S2d).

### Evaluation of potential confounders

Next, we explored the relationship between biopsy tissue site and tumor purity (two key potential confounders) with clinical response across our three modules. Tumor purity was not seen to be predictive of response to therapy using logistic regression in either Bx1 (*P*-value = 0.210) or Bx2 (*P*-value = 0.811) (Supplementary Fig. S3a). However, in Bx1, there were suggestive linear correlations with Mod1 (Turquoise; *P*-value = 0.044) and Mod3 (Brown; *P*-value = 0.013), but not in Bx2 (Supplementary Fig. S3b). From this analysis we observed that lymph node biopsies tended to have higher Mod3 (Brown) eigengene values in Bx1 but not in Bx2. Although most (5/8) samples from patients who achieved a response were derived from lymph node biopsies, it was not seen to be predictive of clinical response (logistic regression; *P*-value = 0.155). Similarly, the module eigengenes were not significantly different by Welch’s T-test between patient samples derived from lymph node vs those derived from other tissues (Supplementary Fig. S3c). Therefore, based on this data, purity and tissue seemed to have limited impact on clinical outcomes and co-expression modules.

### Predicting AMTEC patient response using Mod3 (Brown)

First, we examined the pattern of the eigengenes (PC1 scores) for the three modules in AMTEC with respect to the corresponding Burstein CLIA calls and clinical response (Supplementary Fig. S4). From this comparison, both Mod2 (blue) and Mod3 (brown) had low values for PD patients and patients with Burstein BLIS CLIA calls in both biopsies. For most patient samples, Mod2 and 3 were overall increased for SD or PR patients in Bx2. On the other hand, Mod1 (turquoise) had a mixture of high and low scoring patient samples in Bx1 though they were consistently increased in PD patients for Bx2.

**Fig. 3:**
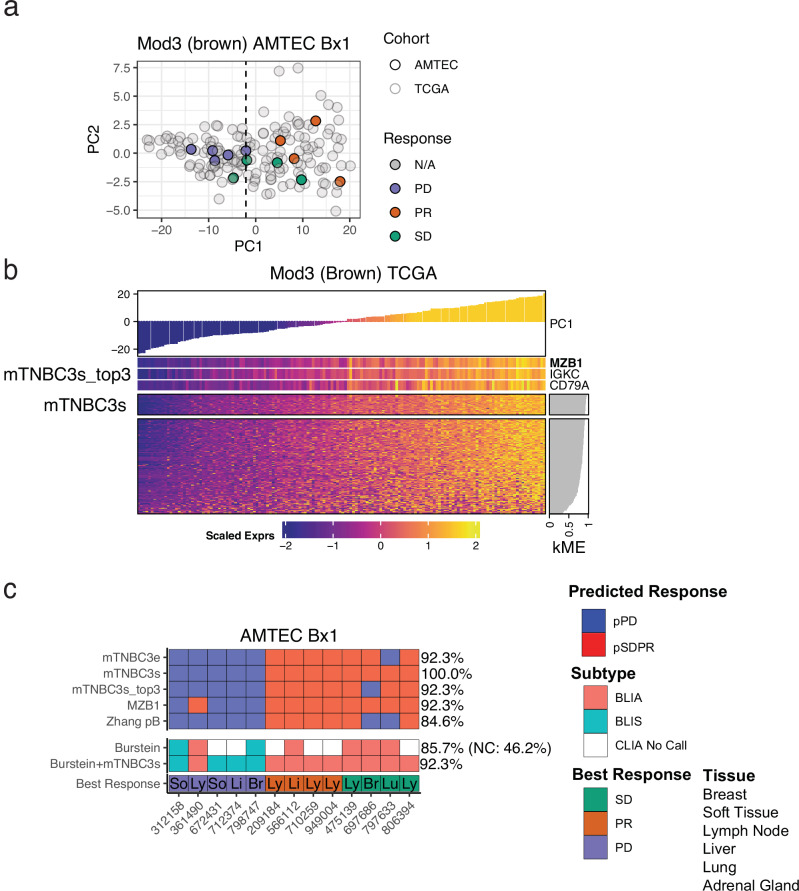
Co-expression module 3 (Brown) is highly prognostic in the AMTEC cohort. **a** Predicted AMTEC module PC1 and PC2 values for Bx1 overlayed on estimated Basal classified BRCA TCGA PC1 and PC2 values for Mod3 (Brown). Low values of the eigengenes (PC1) of Mod3 (Brown) are associated with poor response (progressive disease; PD), while higher values are associated with partial response or stable disease (SD/PR). The dashed lines indicate the conditional inference tree categorization of low vs high for the pre-treatment biopsy (Bx1). **b** The correlation to the module eigengene (kME) provides an unbiased way to rank genes based on their similarity to the expression pattern of the module eigengene. Genes with high kME are likely to be effective for clinically relevant rank-based scoring. Shown is a heatmap of gene expression from the TCGA cohort of all 190 genes in Mod3 (Brown) compared to the eigengene (PC1) values (top), ranked by their kME (right side). The gene sets used for rank-based testing are indicated on the left, as well as the gene with the highest kME, MZB1, in bold. **c** Classifications/predictions for each method (rows) are shown along with the resulting accuracy as a percentage and CLIA No Call rate (NC) if applicable. Accuracy is relative to predicting patient response (PD vs SDPR). The best clinical response is shown at the bottom with the first two letters of the sample tissue overlayed. All of the methods had higher accuracy than that achieved by considering the lymph node as a predictor (71.5%).

Next, we compared the ability of the three expression module signatures to separate PD from SD or PR patients using only their module eigengenes in Bx1. Mod1 (turquoise) and Mod2 (blue) both showed poor accuracy (61.5%, MCC: N/A, F1: N/A), Mod3 (Brown), however, was of particular interest because it achieved 92.3% accuracy (MCC: 0.854, F1: 0.909) in the main AMTEC cohort (Fig. 3a, Supplementary Fig. S1). For Bx2, for Mod1 (turquoise) again had poor performance in the main cohort while both Mod2 (blue) and Mod3 (brown) had increased accuracy (84.6% (MCC: 0.732, F1: 0.833) and 100% (MCC: 1.0, F1: 1.0), respectively; Supplementary Fig. S1). Based on its promising performance for both biopsies in the AMTEC cohort as compared to Mod1 and Mod2, and well as the desire to use this for longitudinal monitoring, we focused on Mod3 (brown) and termed the classifier based on its eigengene mTNBC3e.

However, one challenge with using eigengene-based classifiers is the relative difficulty of externally validating co-expression subnetwork results due to differences in assay platforms and annotation. To increase applicability, gene sets like the MSigDB Hallmarks^11^ are derived from consistently expressed gene sets. Therefore, one approach to validating expression modules would be to transform them into distinct and consistently expressed gene sets and use a rank-based method such as single-sample GSEA^21^ (or alternatively GSVA^22^) to score each sample with respect to each module. In this manner, we leveraged the kME to choose two smaller sets of genes which could be used in more robust rank-based approaches (Fig. 3b). The first of these gene-sets was formed by keeping the thirty-four genes with a kME greater than 0.9, which we referred to as mTNBC3s while the second gene-set used only the top 3 genes (MZB1, IGKC, and CD79A) by kME (termed mTNBC3s_top3). We scored samples using single-sample GSEA and learned the optimal score to separate non-responders (PD) from responders (SD/PR) (see **Methods**). We found that resulting classifiers based on either of the two gene-sets had the same or better performance as using the mTNBC3e (Fig. 3c). The accuracy for mTNBC3s was 100% (MCC: 1.0, F1: 1.0) and for mTNBC3s_top3 was 92.3% (MCC: 0.854, F1: 0.909).

Finally, in addition to using the eigengene as the module representative, often the gene with the highest kME can be considered a module hub gene while maintaining similar predictive performance^23^. In this case, using the centered and scaled expression of only the MZB1 gene maintains similar accuracy (92.3%, MCC: 0.843, F1: 0.889) as mTNBC3e (Fig. 3c).

Given the strong B cell context of Mod 3 (Brown), we assessed the overlap with other similar published signatures. B-cells have been proposed to be a strong predictor of response to either chemotherapy or anti-PD-L1 + chemotherapy^24^. Sub-clustering of the Zhang et al.^24^ single-cell data led to the formation of four main B-cell type signatures (pB, Bfoc, BN, and Bmem). Of these, 3/4 genes in the pB gene-set overlapped with genes in Mod3 (Brown). Using the scoring methodology described in Zhang et al.^24^, we observed moderate performance in the AMTEC cohort (Accuracy: 84.6%, MCC: 0.732, F1: 0.833) but lower than our mTNBC3s series classifiers (Fig. 3c).

A key question was if the network-based signatures could “rescue” the patient samples with Burstein indeterminate subtypes (CLIA “No Calls”), allowing these patients to be classified to aid in therapeutic clinical trial decision aids. Utilizing the mTNBC3s predictor to assign predicted progressive disease calls (pPD) as BLIS and predicted responders (pSD/PR) as BLIA for the indeterminate samples resulted in a subtype/classification approach with high accuracy for either biopsy (Bx1 Accuracy: 92.3%, MCC: 0.843, F1: 0.889; Bx2 Accuracy: 100%, MCC: 1.0, F1: 1.0) and allowed us to provide calls for the remaining 46.2% of the ATMEC samples that were classified as “No Calls” (Fig. 3c).

### Validation of B-cell related classifiers

**Fig. 4:**
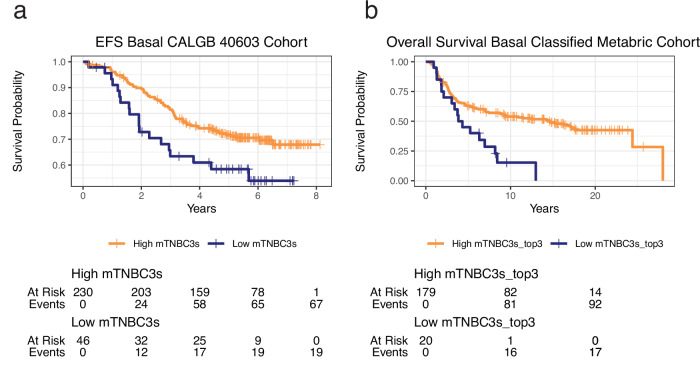
Validation of prognostic B-cell biomarkers. **a** Using the high/low threshold learned from the AMTEC cohort, mTNBC3s was able to significantly differentiate (*P*-value = 0.035; univariate CoxPH; *n* = 276) the CALGB patients based on event-free survival (EFS). Unfortunately, only 5 of the 34 genes in mTNBC3s were available in the METABRIC microarray expression dataset so it could not be screened directly. **b** However, the high/low threshold from mTNBC3s_top3 was further able to identify a 20 patient cohort with significantly lower overall survival (*P*-value = 0.001; logRank; *n* = 199) in Basal-classified samples from METABRIC.

Four of the five classifiers trained on the AMTEC data, including the Zhang’s pB signature, were able to predict patient response in our hold-out validation cohort with high accuracy (100% for Bx1, MCC: 1.0, F1: 1.0), MZB1 was the exception (Accuracy: 80%, MCC: 0.612, F1: 0.857). Three of the five achieved 80% accuracy (mTNBC3s and mTNBC3s_top3 MCC: 0.612, F1: 0.667; Zhang’s pB MCC: 0.667, F1: 0.8) for Bx2. Again, MZB1 as well as mTNBC3e only achieved 60% accuracy (MCC: N/A; F1: N/A) on Bx2. Due to the performance in the pre-treatment biopsy of AMTEC, we believe that these classifiers are potentially prognostic and do not appear to predict response to the AMTEC therapy. To further explore the generalizability of these classifiers as potential prognostic markers, we performed validation in two external breast cancer datasets. The CALGB 40603 dataset consisted of 389 locally advanced TNBC patients who received neoadjuvant chemotherapy with pre-treatment RNASeq samples^20^. Using a 277 patient subset that were classified as having Basal subtypes, we found that classifications based on MZB1, mTNBC3s and mTNBC3s_top3 were significantly associated with pathological complete response (pCR) in the breast (logistic regression; OR: 2.097, 95% CI: 1.274–3.479, *P*-val: 0.004; OR: 2.315, 95% CI: 1.217–4.526, *P*-val: 0.012; OR: 1.918, 95% CI: 1.109–3.353, *P*-val: 0.021; respectively; Supplementary Data S2a). The mTNBC3s classifier was further able to significantly differentiate patients based on event-free survival (single-predictor CoxPH; HR: 0.577, 95% CI: 0.346–0.961, *P*-val: 0.035) (Fig. 4a; Supplementary Data S2a). We noted that the mTNBC3s_top3 was not significant based on the AMTEC data cutoff for high vs low (single-predictor CoxPH; HR: 0.705, 95% CI: 0.445–1.117, *P*-val: 0.136; Supplementary Data S2a). However, single-predictor Cox Proportional Hazards models for both mTNBC3s and mTNBCs_Top3 were significant (HR: 0.311, 95% CI: 0.120–0.804, *P*-val: 0.016; HR: 0.439, 95% CI: 0.206–0.936, *P*-val: 0.033; respectively) without binning the actual scores; highlighting the potential for further refinement of the cutoff value (Supplementary Data S2a).

Although mTNBC3s couldn’t be tested in Basal-classified METABRIC^25^ patients as only 5/34 genes were present in the processed microarray data, mTNBC3s_top3 was able to differentiate a subset of 20 patients having poor overall survival (*P*-value = 0.001; logRank; Fig. 4b; Supplementary Data S2b). In addition, it remained significant after adjusting for clinical covariates in the Cox proportional hazards model discussed previously^25^ (Cox PH Likelihood ratio test; *P*-value = 0.009; Supplementary Data S2c). These results indicated that the network-based mTNBC3 and mTNBC3_top3 classifiers, although originally derived in the context of metastatic TNBC, are potentially prognostic and generalizable beyond their original context.

### Patient-specific network measures predict best response

**Fig. 5:**
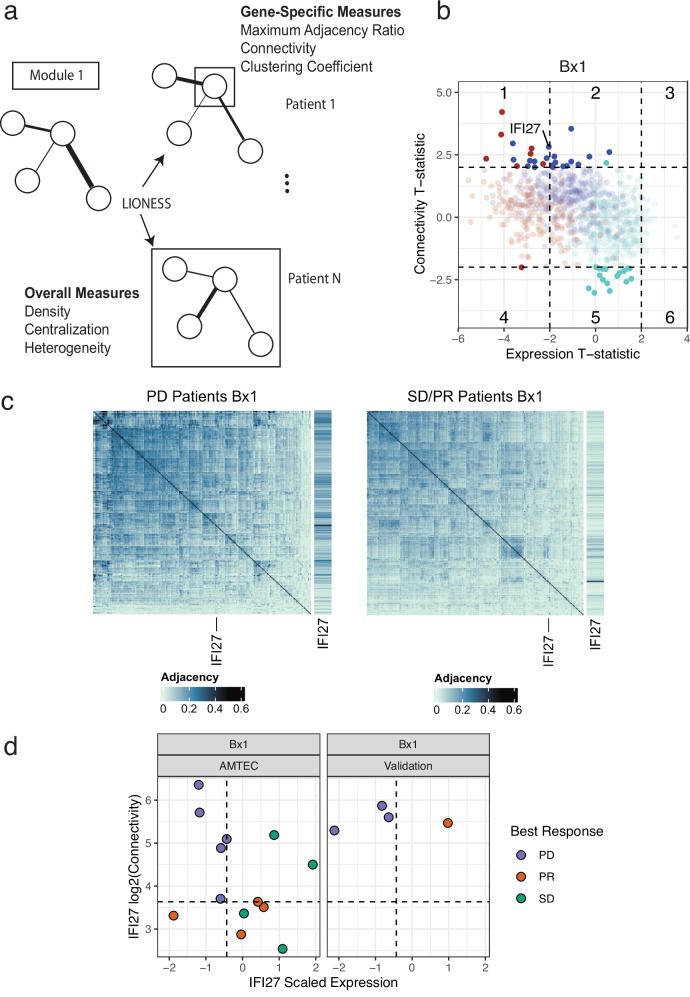
Patient-specific subnetwork rewiring predicts response. **a** The WGCNA co-expression modules were decomposed into patient-specific weighted (sub)networks using LIONESS. Each subnetwork was scored using overall metrics such as Network Centralization, as well as gene-specific metrics such as Connectivity. **b** Comparing average differences in Connectivity between responders and non-responder patients (Y-axis) to average differences in expression (X-axis), we see that the majority of differences can be attributed solely to expression changes. Dots are colored by module. We highlighted genes where differences could be attributed solely to changes in Connectivity (Sectors 2 and 5) as well as those where potentially a combination of expression and Connectivity differences could be more predictive of response (Sectors 1, 4, 3, and 6). IFI27 in Sector 1 was an example of a gene that had both a patient-specific expression and a Connectivity component related to response, but would not have been detected in traditional DE comparisons. **c** Comparing the average gene connections (termed Adjacencies) with respect to all 323 genes in Mod2 (blue) there is a marked decrease in Adjacencies between non-responder patients (PD) and responder patients (SD and PR) for Bx1. For each plot, a blown-up version of IFI27 is shown on the right. **d** As the Connectivity is the sum of Adjacencies for a given gene, comparing Connectivity and expression for IFI27 shows that a combination of high connectivity and low expression separates non-responder patients from those who did achieve a response.

To realize the full utility of network medicine and provide informative readouts for precision oncology tumor boards, patient-specific networks are key to assess individual baseline and response to treatment. In order to explore individual changes in wiring, we adapted the LIONESS approach^26,27^ to further decompose each of the 3 co-expression modules of interest into their estimated patient sample-specific subnetworks based on interpolation from the reference cohort. This resulted in 78 total subnetworks. For each subnetwork we computed a set of gene-specific summary measures including Connectivity, the Maximum Adjacency Ratio (MAR), and the Clustering Coefficient^28^ (Fig. 5a). Adjacency and Connectivity are fundamental network measures with Adjacency defined as the strength of the connection/association between two genes ranging between 0-1, whereas Connectivity is defined as the per-gene sum of the Adjacencies to all the other genes (i.e., sum of its connection strengths with all other genes in the network). We also computed overall measures including Density, Centralization and Heterogeneity, metrics that could quantify potential re-wiring at the module level^28^ (Fig. 5a). However, in AMTEC, these measures were not predictive of response so only the gene-specific network features (Connectivity, MAR, and Clustering Coefficient) were considered further.

Given the strength of the observed expression differences in Mod3 (Brown), we were interested in determining if these gene-specific network measures provided additional information beyond simply reflecting overall differences in expression. We first carried out a differential expression analysis between responders (SD/PR) and non-responder patients (PD) separately for each biopsy. Next, we carried out a similar analysis for each of the three gene-level network features. We visualized the T-statistics from these results using ‘sector’ plots^23^. In these plots, sectors 2 and 5 indicate differences due only to sub-network differences, while sectors 1,3,4, and 6 indicate genes which may have both an expression and network component (Supplementary Fig. S5a). Focusing on the latter sectors for Connectivity we found that the IFI27 gene from Mod2 (Blue) was one of the top genes (not in Mod3 (Brown)) for Bx1 (Fig. 5b). Examining the average Adjacency matrices for Mod2 (Blue), we see that there is a clear decrease in connection strength for the responders (SD/PR) relative to non-responders (PD) for IFI27 (Fig. 5c). Note that no genes had significantly different expression in Bx1, but 77 were found in Bx2 (FDR < 0.05; Supplementary Data S3a). Similarly, no genes had significantly different network features for either biopsy (Supplementary Data S3b). Interestingly, for IFI27, a combination of expression and connectivity provided separation of responders from non-responders in both AMTEC and the Validation cohort (Fig. 5d).

### Temporal differences in gene-level network measures predict best response

**Fig. 6:**
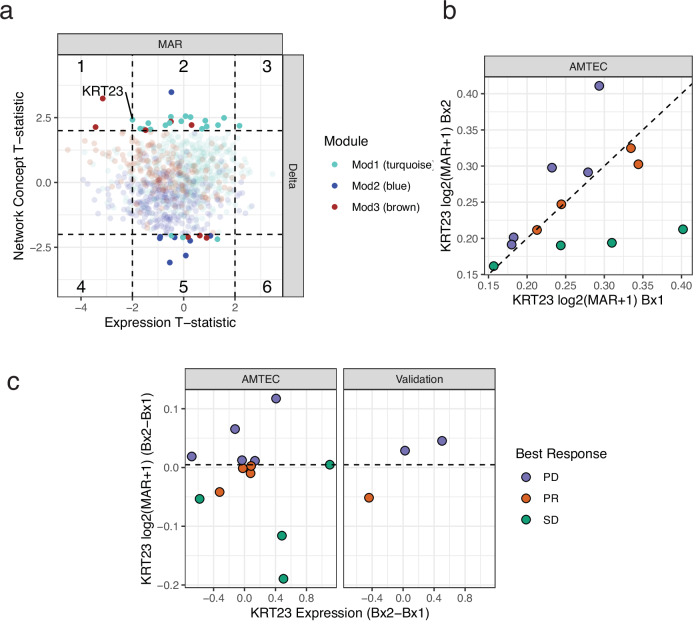
Temporal differences between KRT23 subnetwork-based MAR values predict response. **a** Shown is a sector plot of T-statistics from the average differences between Bx2-Bx1 (termed Delta) with respect to best response for the Maximum Adjacency Ratio (MAR) network feature. KRT23 is a member of sector 2 indicating that the average difference in MAR delta was more associated with response than the corresponding expression delta. **b** Comparing KRT23 MAR values between Bx2 and Bx1 showed that PD patients tended to have higher values in Bx2 than SD or PR patients. **c** This small increase in MAR between Bx2 relative to Bx1 (dashed line) is sufficient to separate PD from SD or PR patients independent of expression.

Given the paired biopsy nature of the study, we were interested in assessing the informativeness of the temporal differences in expression and network features. We performed differential expression and network-wiring testing to determine whether the average differences between Bx2 and Bx1 were different between responders (SD/PR) and non-responders (PD). Again, we used sector plots to visualize the relationship between expression and the network features (Supplementary Fig. S5b). The KRT23 gene relative to the EMT-related Mod1 (turquoise) was one of the top genes in sector 2 for the Maximum Adjacency Ratio (MAR) network measure (Fig. 6a). A small increase in MAR was seen in Bx2 samples that could differentiate responders (SD/PR) from non-responders (PD) (Fig. 6b). Independent of expression, KRT23 MAR was able to achieve separation of responders (SD/PR) from non-responders (PD) patients in both AMTEC and the validation cohort (Fig. 6c), warranting further examination of patient specific temporal network measures in other studies. Note that no genes had significantly different expression or network features (FDR < 0.05; Supplementary Data S4a,b) indicating the potential utility of this approach for identifying patient-specific temporal changes as they would not have been identified through traditional approaches.

## Discussion

Based on initial analysis of the AMTEC cohort, which indicated that one of the most informative predictors of response in TNBC was the expression-based Burstein molecular subtypes, we further expanded on these data through network-based analyses. In the CLIA environment, when the calls were definitively BLIA or BLIS, they were highly predictive. However, there were a number of samples that did not have a strong signal that identified a sample as either BLIA or BLIS and were thus considered “Indeterminant (IND)” and assigned a “No Call” label. Our network-based analysis was used to identify approaches to improve the clinical utility of the Burstein molecular subtypes.

Three out of the five co-expression modules learned from Basal-classified TCGA BRCA patient reference cohort were significantly enriched for a MSigDB Hallmarks. Based on this annotation as well as orthogonal multi-modal support of these modules (RPPA and mIHC), we attributed Mod1 (Turquoise) to EMT/cell adhesion, Mod2 (Blue) represented immunomodulatory and Mod3 (Brown) reflected plasma/B-cell processes.

Immune repertoire profiling of the AMTEC cohort RNASeq data further reinforced the idea that the eigengene from Mod 3 (Brown) tracks the pattern of relative expression of B-cell-related genes, especially immunoglobulin genes. Harris et al.^29^ found that tumor-infiltrating B lymphocyte-enriched tumors showed preferential clonal expansion of IgG isotypes and were associated with improved clinical outcome^29^. Similarly, Shephard et al. 2022 saw reductions of IgG diversity, defined as low Evenness, in TNBC patients achieving a clinical response. They concluded that this potentially indicated clonal expansion and Ig class switching associated with a directed immune response^20^. However, we saw no significant difference in the Evenness measure between patients with and without a clinical response. This is likely due to several factors. For instance, our cohort size was small and might not be able to detect a subtle effect. Our study focused on metastatic as opposed to primary TNBC. Additionally, patients who did not achieve a response tended to have low abundance of the immunoglobulin chains. This, in turn, would lead to poor sampling of available clonotypes, potentially contributing to observed low diversity in the un-normalized measures such as Shannon’s entropy, which disappears after normalizing to form the evenness measure. This can only be corrected to a certain point using down-sampling, since down-sampling results in further information loss. Finally, there were differences in protocols, such as the use of a different alignment and post-processing pipeline. Importantly, we discarded samples that had too little data to be informative. Considering samples with low representation as having high evenness had the potential to artificially create differences in evenness, especially in our clinical trial dataset.

The Mod2 (blue) module demonstrated superior ability in distinguishing between BLIA and BLIS samples within the TCGA dataset, outperforming both Mod3 (brown) and Mod1 (turquoise). Despite its effectiveness in TCGA, predictive models derived from Mod2’s eigengene showed less temporal consistency compared to Mod3 in AMTEC. Mod2 demonstrated predictive capability only in the second biopsy (Bx2), while Mod3 had high predictive performance in both biopsy timepoints. Mod1 was not considered further as it exhibited low predictive value across both biopsy timepoints.

Predictive models formed from the plasma/B-cell related Mod3 (Brown) co-expression module mTNBC3e, as well as more clinically accessible mTNBC3s versions, achieved excellent classification accuracy with separating responders (high values) from non-responders (low values) in AMTEC and our holdout Validation cohort. When applied to the CALGB 40603 clinical cohort and METABRIC, the mTNBC3s classifiers were significantly associated with pathological complete response and event free survival in CALGB and identified a subset of 20 patient samples with poor response in METABRIC, highlighting that this signature had prognostic activity and was not directly related to response to a specific therapy. However, it remains possible that there could be an additional component of predictive value for specific therapies. A single-cell RNASeq experiment of tumors from TNBC patients treated with paclitaxel or paclitaxel in combination with atezolizumab found that B-cells were the most predictive immune cell type for patients achieving a response^24^. However, they found that follicular B-cells (Bfoc) were the most important B-cell subset for their cohort. Interestingly, of the four B-cell subsets evaluated in the AMTEC samples, plasma B-cells (pB), not Bfoc, were the strongest predictor of response. We were able to independently derive biologically similar small gene-sets from bulk RNA sequencing using co-expression-based network methodology, highlighting the potential value of generating and re-analyzing existing large datasets with network-based approaches.

The plasma and B-cell related mTNBC3s signature was derived from an adjuvant study (TCGA) and independently validated in neoadjuvant (CALGB) studies as well as in our AMTEC metastatic cohort. In addition, it was independently validated in METABRIC. This suggests a potentially generalized utility related to prognosis and the potential that the gene signature may identify metastatic potential as well as aggressiveness of metastatic tumors that determine the overall outcomes in TNBC.

Despite the AMTEC cohort comprising patients with metastatic breast cancer—a population historically associated with poor survival outcomes—the development of prognostic or predictive biomarkers retains critical clinical relevance. Such markers could enhance therapeutic decision-making by identifying subgroups likely to derive sustained benefit from stratified treatment approaches. As noted in a recent evaluation of biomarkers for immune checkpoint inhibitors (ICIs) in advanced melanoma, robust prognostic risk stratification can guide more precise utilization of ICIs to reduce over-treatment^30^. Furthermore, prognostic biomarkers may serve dual purposes within the broader framework of Awareness of Disease Status (ADS) facilitating earlier transitions to palliative care when appropriate, allowing patients and providers to align care plans with clinical trajectories and personal priorities^31^.

In addition to serving as a stand-alone predictor, the plasma and B-cell related mTNBC3s signature also augmented the Burstein BLIA-BLIS CLIA calls, resolving indeterminate (No call) samples. This immediately extends the utility and applicability of this approach.

It is important to note that there are limitations to this study. The first is the small sample size of the primary clinical cohort, a known challenge in precision oncology trials focused on in-depth longitudinal characterization. This is ameliorated to some degree by the use of large public datasets and orthogonal data to help validate our findings. Another potential limitation was the use of non-metastatic patients from TCGA to build the initial network. While this could impact generalizability to the metastatic patient population, we instead found that the TCGA-based eigengene and geneset signatures are predictive in our metastatic cohort. This highlights the potential preservation of prognostic gene expression profiles between primary and metastatic disease, which has been previously observed^32^. This preservation is not entirely unexpected, as long-term patient outcomes often depend on the development and aggressiveness of metastases, given that primary disease is typically well-controlled with current therapeutic approaches.

In addition to the evaluation of our WGCNA co-expression module eigengene-based predictors, we also formed patient-specific subnetworks based on the module genes. We showed that features derived from these patient-specific subnetworks could potentially be used as prognostic or predictive biomarkers, both in conjunction with gene-expression or without. However, given sample size constraints, the clinical utility of these signatures and this approach in general is yet to be determined. Our network-based approach provided many benefits over traditional differential expression. For example, neither the standard paired T-test between biopsies, the test of paired differences, nor Bx1-only samples between patient response groups provided a significant result after FDR adjustment. Only the comparison of Bx2 samples between response groups provided significant differential expression. These 77 DE genes included both MZB1 and IGKC but not CD79A, which make up mTNCBC3s_top3. However, IGKC was ranked 51/77, and MZB1 was ranked 58/77 based on fold change. These genes would not have been associated together using standard differential expression analysis, highlighting again the strength of network-based predictors. In addition, we highlighted the informativeness of patient-specific network perturbations, which also would be missed (e.g., IFI27 and KRT23 were non-significant in the traditional DE comparisons as well). By leveraging the highly correlated subnetworks from WGCNA, we were more easily able to identify the most prominent biological themes. We were then able to use multiple orthogonal approaches to identify both expression and co-expression subnetwork patterns potentially associated with patient prognosis and response.

## Methods

### Reference and clinical cohort

To allow assessment of the degree of therapeutic changes in on-therapy clinical trial samples, we utilized 152 Basal-classified Breast Cancer samples from The Cancer Genome Atlas (TCGA) Project^7^ as our “reference cohort”. For TCGA, Institutional review boards at each tissue source site reviewed protocols and consent documentation and approved submission of cases to TCGA^7^. For our clinical cohort, all patients gave informed consent to participate in this study, which had the approval and guidance of the Institutional Review Board at Oregon Health and Science University (OHSU IRB #18504). All human subjects research was performed in accordance with the Declaration of Helsinki.

For the clinical trial cohort comparator, 13 patients with metastatic triple negative breast cancer (mTNBC) from the phase II clinical trial (NCT03801369; Adaptive multi-drug treatment of evolving cancers (AMTEC))^6,9,10^ each having paired pre-treatment (Bx1) and on-treatment (Bx2) samples, were used (denoted as the “AMTEC cohort”). Additionally, we held out a second group of 10 patient samples (5 Bx1 and 5 Bx2, 3 of which were paired), denoted as the “Validation cohort”. For this study, our main outcome was based on the best response achieved by a given patient as part of the trial. These outcomes were defined as either progressive disease (PD), stable disease (SD), or partial response (PR). For classification, we further binned patients into those who achieved a “best response” (SD/PR) vs those who did not (PD). For external independent validation, we used RNA-Seq from 277 Basal classified patients from CALGB 40603 clinical trial^20^ (re-processed as below), as well as microarray expression data for 199 Basal classified patients from METABRIC^25^.

### RNA sequencing data processing

Kallisto (an RNA-seq quantification algorithm^33^) processed abundance values (Transcripts Per Million, known as TPM) were retrieved for TCGA^34^ and limited to the Basal subtype samples. Fourteen samples were removed due to having relatively low expression, leaving 152 samples.

For the AMTEC samples, preparation of RNA and transcriptome sequencing was performed at the Knight Diagnostics Laboratories. Total nucleic acid was extracted from macro-dissected, tumor-rich areas from FFPE sections, purified, and used for next generation sequencing (NGS). Libraries were prepared using the TruSeq RNA Access library preparation kit and sequenced on the Illumina NextSeq500. Approximately 100 million reads were generated per sample. For both the AMTEC and CALGB cohort samples, gene expression was quantified relative to Gencode v24^35^ transcripts using Kallisto (v0.43)^33^. The AMTEC patient cohort was limited to 13 Bx1 and Bx2 pairs excluding CLIA-classified LAR samples.

### Weighted Gene Coexpression Network Analysis (WGCNA)

Coexpression network modules for the TCGA data were formed using WGCNA (v1.71)^36^ using the top 2000 most variable genes after log2 transformation. A range of parameters were assessed for WGCNA using stability assessment of module assignments using 50 iterations of 63.2% subsampling and assessment of module quality^37^. Topological significance was based on the Z-scores of the density-based measures relative to 100 random gene sets. We required a median Z-score of 2 or greater. The final WGCNA parameters were a signed hybrid network with power of 5 using bicor correlation, deepSplit =2, detectCutHeight = 0.995, minimum module size of 30, and pamStage=TRUE. For comparison with AMTEC and the validation cohort, the abundance values were batch corrected using ComBat from the SVA package (v3.44.0)^38^ using TCGA as the reference batch. Principal component scores were computed for the TCGA cohort after centering and scaling. AMTEC cohort PC scores values were ‘predicted’ using the means, standard deviations, and eigenvectors from the TCGA cohort. Gene set enrichment for the MSigDB Hallmarks was performed using clusterProfiler (v4.4.4)^39^. Benjamini-Yekutieli^40^ false discovery rate (FDR) adjustment was used. Single-sample GSEA was limited to the WGCNA gene universe using GSVA (v1.44.5). The ssGSEA normalization was not performed.

### Immune cell type scoring

The immune cell type analysis followed a prior approach^41^ for calculating immune cell scores. Briefly, Gene Set Variation Analysis (GSVA)^22^ (v1.44.5) was performed on the log2 TPM values from the AMTEC cohort relative to 16 immune cell gene sets^19^ using a Gaussian kernel cumulative density function (kcdf = ”Gaussian”). The GSVA enrichment statistic was calculated as the magnitude difference between the highest and lowest random walk deviations (mx.diff=TRUE).

### Immune Repetroire profiling

We used TRUST4 (v1.0.8)^42^ to assemble BCR and TCR repertoires from AMTEC bulk RNASeq data. As part of the TRUST4 post-processing, we defined BCR clonotypes by clustering CDR3 sequences after matching on length, and assigned V and J genes with a cutoff of 0.8. TCR clonotypes were based on the CDR3 sequence only. To define the IGH and IGHG classes, we used the distinct clonotypes defined for each of the isotypes. For each class, we computed several diversity measures metrics to assess the variety and distribution of different types or entities within the group) including Shannons’ Entropy, Evenness (normalized Shannon’s Entropy, which is a measure of how evenly distributed the entities are), D50, Gini-Shannon as well as the Gini Coefficient. Diversity measures were examined both in the original values as well as after downsampling reads to 8500 for Bx1 and 400 for Bx2, levels that ensured at least three PD patients would remain after excluding samples lower than the corresponding thresholds. Downsampling was repeated five times, with the median used for comparison between groups. Missing values were removed prior to testing.

### Patient-specific networks

We used the Linear Interpolation to Obtain Network Estimates for Single Samples (LIONESS) method (v1.10.0)^43^ to generate patient-specific sub-networks for each AMTEC patient sample and gene coexpression module. As LIONESS interpolates networks based on gene correlation values for a single cohort, we combined each AMTEC patient sample with the TCGA cohort in turn to compute the sample’s network. We converted each subnetwork to a standard adjacency network by removing negatively weighted edges and scaling the remainder to be between 0 and 1. The WGCNA function ‘fundamentalNetworkConcepts‘ was used to compute the overall and gene-specific network features. Differential network feature analysis was performed using limma (v3.52.4)^44^ after log2 transformation.

### Classification

Either logistic regression or the Conditional Inference Tree (ctree)^45^ methodology was used for the best response classification as indicated in the text. We used the ctree implementation from the ‘partykit‘ R package (1.2-16). Single variable models required ‘minbucket‘ and ‘minsplit‘ to be 3, limiting depth to 1. The Zhang et al.^24^ genesets were scored by averaging the expression values across the genes, with the high vs low categorization performed using the median of those scores as described in their manuscript^24^. We computed accuracy and the F1 measure using the caret (6.0-94)^46^ R package and Mathews correlation coefficient (MCC) using yardstick (1.2.0)^47^.

### Burstein (BLIA/BLIS/LAR) subtype calling

The Burstein subtypes were computed for TCGA using the refined list of 77 genes^48^. Spearman’s correlation was computed between the gene expression values and each centroid. Patient samples were assigned to the centroid with the highest correlation. Calls were considered indeterminate if the difference between the top two centroids was less than 0.1.

### Differential expression analysis

For the AMTEC cohort, counts were formed from scaled TPM abundance using the tximport (v1.24.0) package. The limma-trend pipeline (limma v3.52.4 and edgeR v3.38.4) was used for model fitting^49^, including TMM normalization^50^.

### Reverse phase protein array

The TCGA Processed RPPA data was downloaded from The Cancer Proteome Atlas (TCPA). Data had been already been standardized and normalized by TCPA as described previously^51^.

### Multiplex immunohistochemistry

For the AMTEC cohort, multiplex immunohistochemistry (mIHC) was performed as previously described^52^. Cell phenotypes were assigned with hierarchically gating and quantified to cell densities (cells/mm^2^). Cell phenotype densities were log10 transformed.

### Statistics and reproducibility

All analyses were carried out using R v4.3.1. Visualizations were generated using ggplot2 v3.5.0^53^ or ComplexHeatmap v2.16.0^54^. All *P*-values are reported unadjusted unless otherwise specified.

## Supplementary information

Supplemental Figures S1-5 and Supplemental Data legends

Supplemental Data S1

Supplemental Data S2

Supplemental Data S3

Supplemental Data S4

Supplemental Data S5

Supplemental Data S6

## Data Availability

All data is available through the HTAN Data Portal as part of the HTAN OHSU Atlas (https://data.humantumoratlas.org/). Mapping to HTAN patient identifiers is provided in Supplementary Data S5. Raw sequencing data have been deposited in dbGAP (Project phs002371.v1.p1). RPPA data was from TCPA^55^. METABRIC data was downloaded from CBioPortal^56^. CALGB RNASeq data were retrieved from SRA through dbGaP (phs001863.v1.p1). The mIHC data is provided in Supplementary Data S6.

## Code availability

The code (and corresponding parameters) to reproduce manuscript results is freely available in our GitHub repository under a GPL-3.0 license at: https://github.com/biodev/amtec_manuscript.
