# Supplementary material for: Utilizing cohort-level and individual networks to predict best response in patients with metastatic triple negative breast cancer
Source: NPJ Precis Oncol. 2025 Jun 13;9:179. doi: 10.1038/s41698-025-00959-w (PMC12166044; doi:10.1038/s41698-025-00959-w)
Supplement: Supplementary file 1 — Supplemental Figures S1-5 and Supplemental Data legends [file 41698_2025_959_MOESM1_ESM.docx]

**Figure S1. AMTEC module PC scores overlap with TCGA-BRCA highlighting the validity of our network-based approach.** Predicted module PC1 and PC2 scores from both the AMTEC cohort (left side) and the Validation cohort (right side) were overlayed on estimated Basal classified BRCA TCGA PC1 and PC2 values and colored by best response. The overlap of AMTEC within TCGA further confirms the appropriateness of TCGA-BRCA as the basis of network inference. The dashed lines indicate the categorization of Low vs High based on significant (P-value < .05) splits from a conditional inference tree model fit on each module eigengene in the AMTEC cohort.


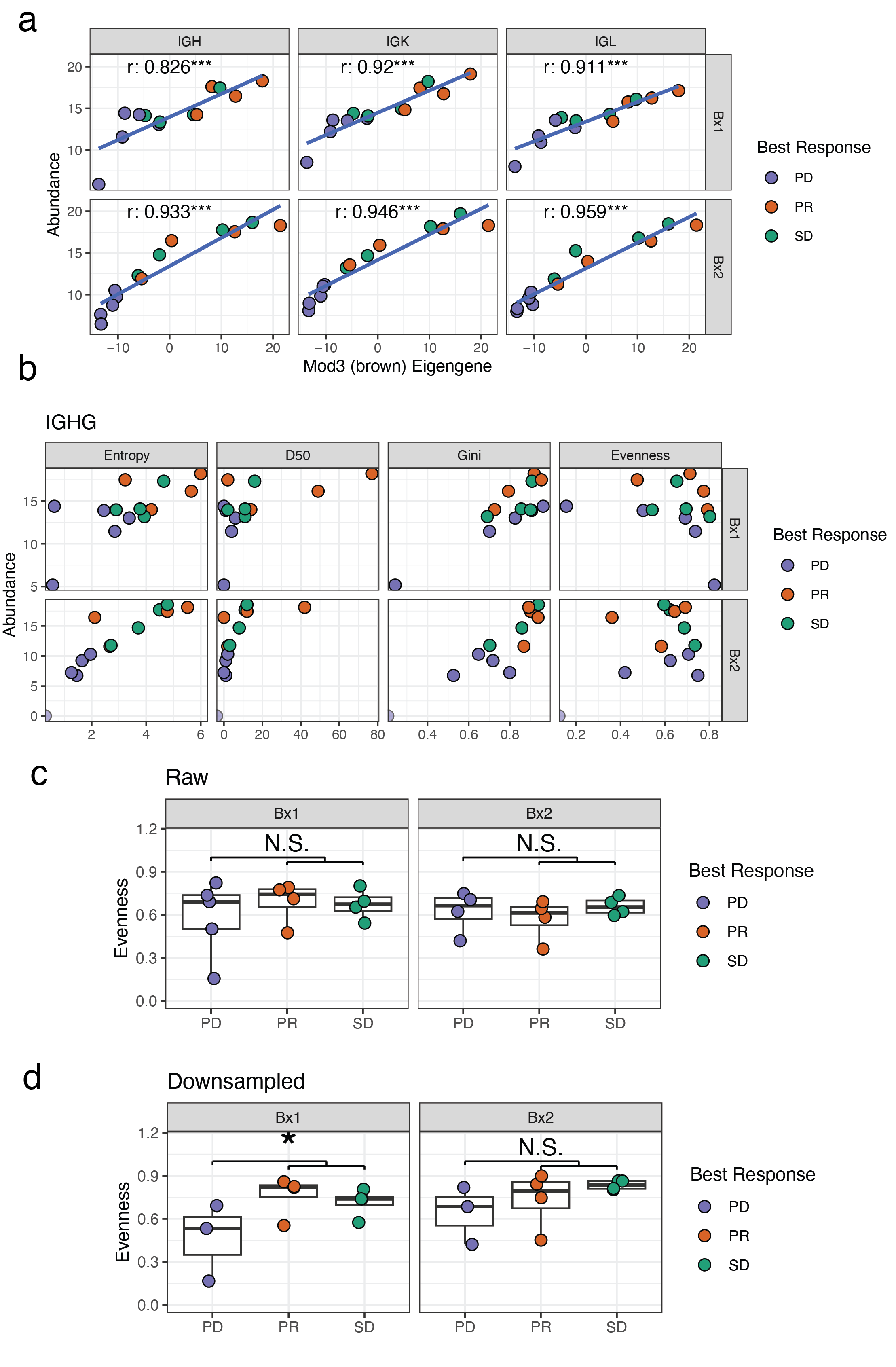


**Figure S2. Assessment of immune repertoire in AMTEC.** (**a**) Module 3 (brown) eigengene has a significant Pearson’s correlation with the abundance (log2 read count) of immunoglobulin chains in both Bx1 and Bx2 (n=13). (**b**) Limiting clonotypes to the immunoglobulin G class, low abundance appeared to be associated with decreases in diversity measures for the patients who did not achieve a response (PD) relative to those who achieved stable disease or partial response (SD/PR). The exception to this was the Evenness measure which is normalized Entropy. (**c**). No differences in Evenness values were seen between PD patients relative to those achieving SD or PR in either biopsy (Wilcoxon rank sum test; P-values=0.724, 0.570; n=13). We further attempted to reduce any effect due to the differences in the quantity of reads and clonotypes between samples by down-sampling reads for the IGH chain. (**d**) However, this only showed a marginal difference between PD and SD or PR patients for Bx1 (Wilcoxon rank sum test; P-Value = 0.048; n=11) and no difference for Bx2 (Wilcoxon rank sum test; P-Value = 0.194; n=11). The symbols ‘*’, ‘**’ and ‘***’ indicates unadjusted P-values < .05, .01 and .001 respectively. ‘N.S.’ indicates not meeting significance criteria (P-value < .05).


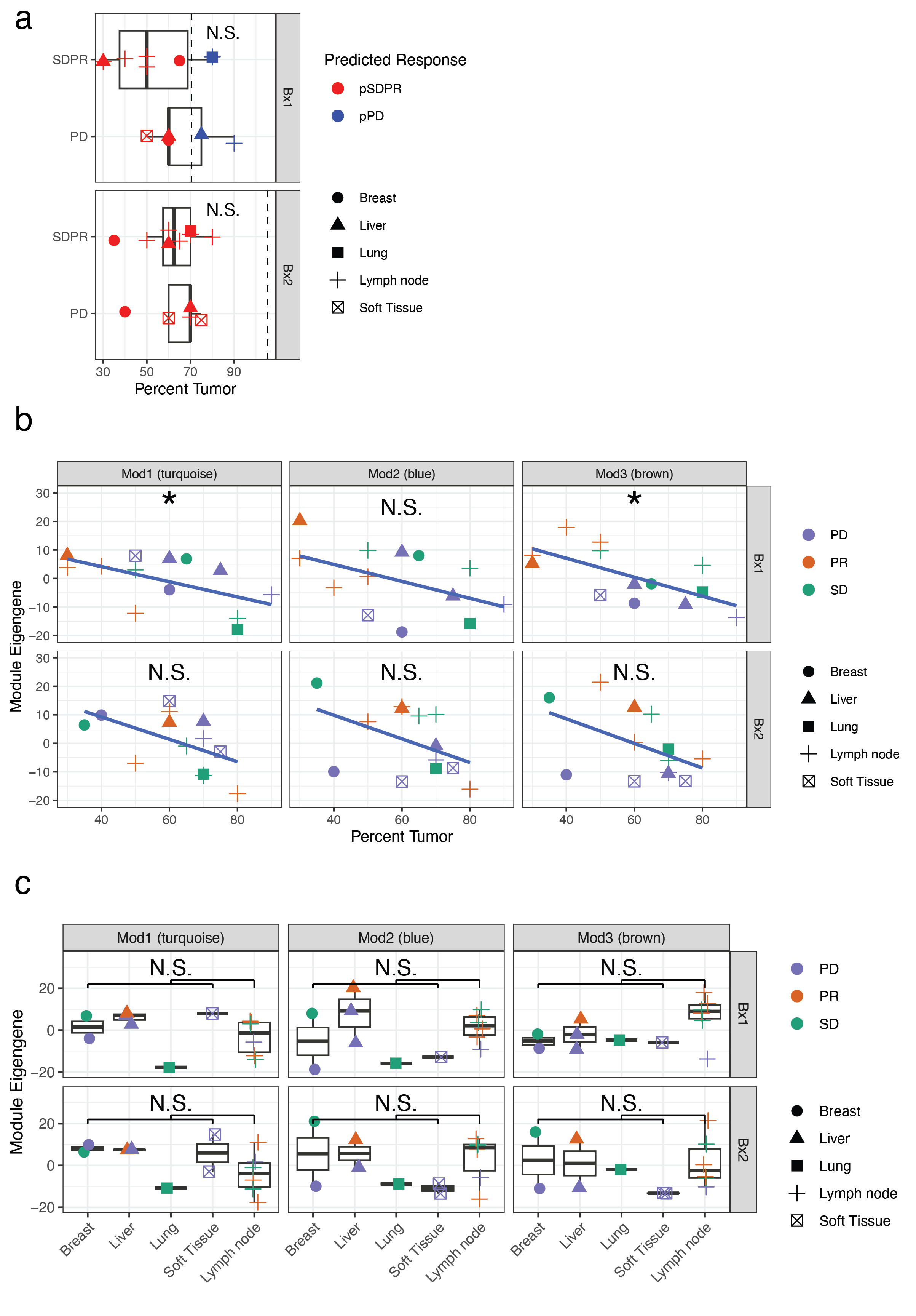
**Figure S3. Tumor purity is marginally associated with module eigengene expression in Bx1 but does not predict response.** (**a**). There was no significant association between percent tumor and clinical response in either Bx1 (logistic regression; P-value=0.210; n=13) or Bx2 (logistic regression; P-value=0.811; n=13). Points indicate patient samples, blue color indicates predicted non-response (pPD), red indicates predicted response (pSDPR). Dashed lines are the decision boundaries. Shapes indicate tissue source. (**b**) Percent tumor was correlated with Mod1 (turquoise)(Pearson’s correlation; P-value: 0.044; n=13) and Mod3 (brown) (Pearson’s correlation; P-value=0.013; n=13) eigengenes in Bx1 but not in Bx2. (**c**) Comparing the module eigengenes between patient samples derived from lymph node compared to other sources is not significantly associated with any module eigengene (Welch’s T-test; n=13). The symbols ‘*’, ‘**’ and ‘***’ indicates unadjusted P-values < .05, .01 and .001 respectively. ‘N.S.’ indicates not meeting significance criteria (P-value < .05).

**Figure S4. Relationship between module eigengenes, Burstein subtypes and clinical response.** For each biopsy a heatmap is shown indicating two types of predictors for the AMTEC cohort: The PC1 scores (eigengenes) for each of the three modules (top) and the CLIA calls for the Burstein subtypes (middle). The best clinical response for each patient (X-axis) is also shown (bottom). Each biopsy is sorted first by the Burstein subtypes and then by clinical response.

a

b


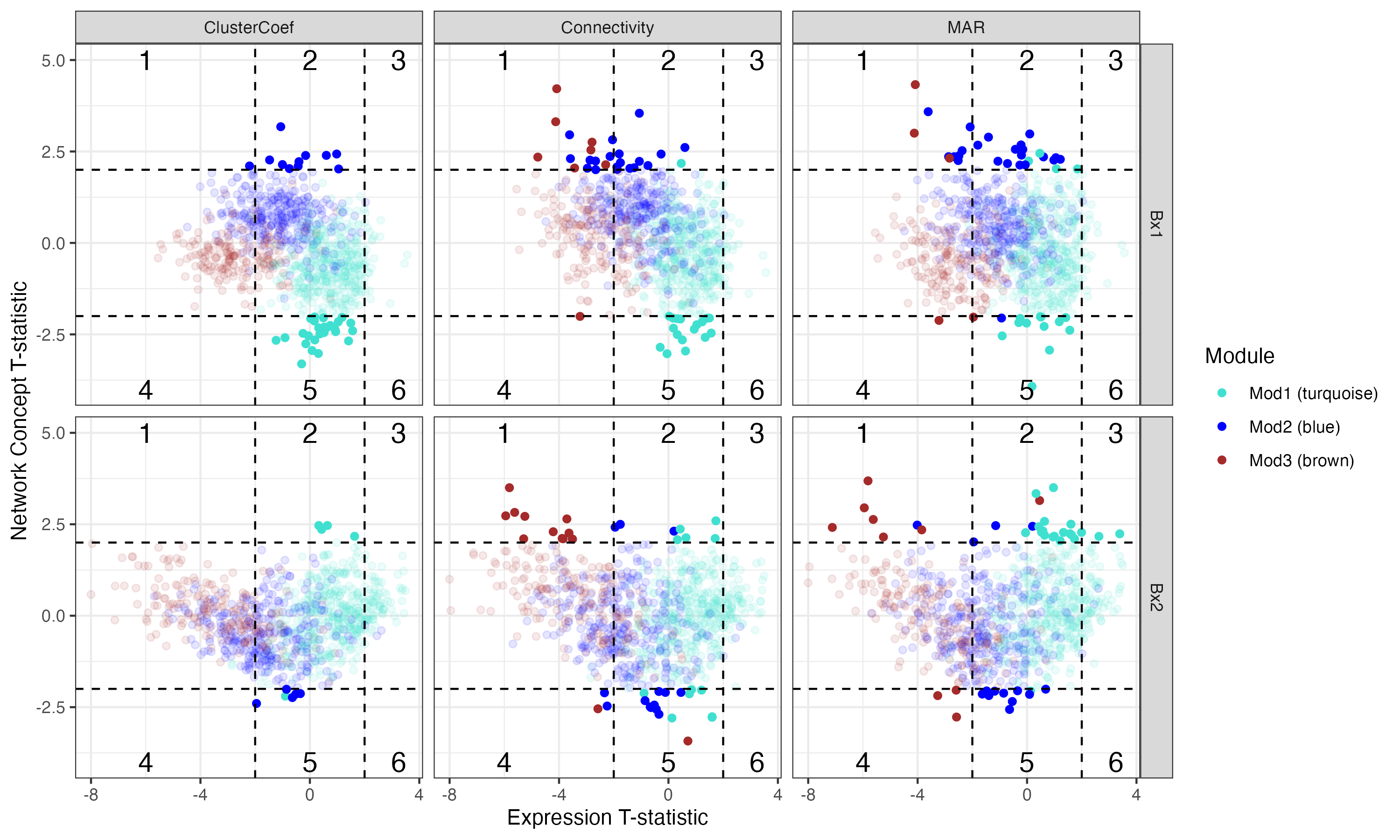

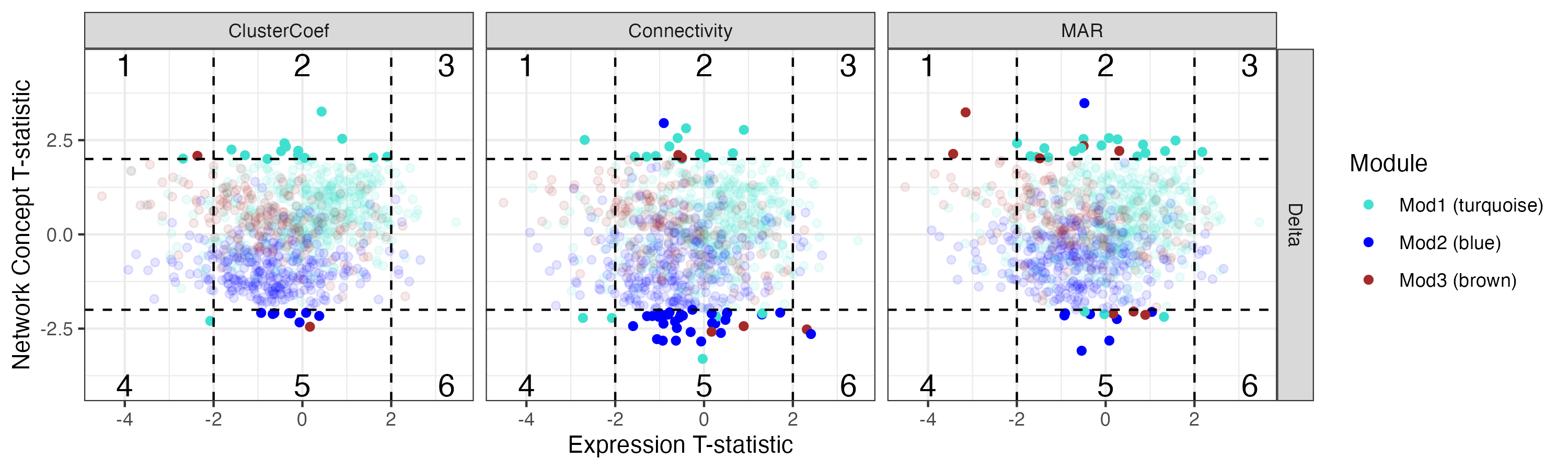


**Figure S5. Relationship between expression and network feature differences in AMTEC.** Sector plots are shown where the gene-level T-statistics represent differences between PD and SD/PR patients for expression (X-axis) and network features (Y-axis). The sectors can be interpreted based on their numbers. For instance, sectors 2 and 5 indicate genes which have a greater difference in a given network feature than expression. Sectors 1, 3, 4 and 6 on the other hand indicate genes which have both differences in expression and network feature. (**a**) Per biopsy (Y-facet) T-statistics are shown while (**b**) indicates T-statistics derived using the ‘Delta’ (Bx1-Bx2). For both **a** and **b**, a plot is shown separately for each network feature (Clustering Coefficient, Connectivity and Maximum Adjacency Ratio left to right respectively). Network features have been transformed using log2(feature + 1).

**SUPPLEMENTAL DATA LEGENDS**

**Supplementary Data S1. Summary of Network Module Membership.** This provides information on the assignment of the top 2000 most variable genes to modules using WGCNA. There is one row per Gencode v24 gene with columns for the specified gene identifier, symbol and gene type. Each module is represented by a number and color (module assignments) which are provided in the module_name column. Note that Mod0 (grey) is the 'unassigned' module and is therefore held out for most analyses. Finally, the correlation with the module eigengene is a metric of module membership (kme) which indicates how much an individual gene is contributing to the overall module behavior.

**Supplementary Data S2**. **Summary of external validation model fits.** (**a**). Results from each signature in the CALGB dataset. Pathological complete response (pCR) was assessed using univariate logistic regression, event free survival (EFS) by a univariate Cox proportional hazards model. (**b**) Results from logRank tests of overall survival in the METABRIC cohort. (**c**) Cox proportional hazards model fits including coefficients for grade, age, lymph nodes examined and tumor size as well as the signature. Highlighted is the signature coefficient as well as the results from a likelihood ratio comparing the full model to one containing only the clinical variables. For (**a-c**) two encodings were used for each signature, ‘Score’ indicates the use of the score as a continuous variable, ‘High vs Low’ indicates use of the corresponding binary categorization learned from AMTEC.

**Supplementary Data S3. Differential expression/network features by biopsy.** (**a**) Differential expression results are provided for each biopsy. (**b**) Differential network feature results are provided per module, feature, biopsy and gene. Each comparison is the average difference in expression (or network feature values) between PD vs SD/PR patients.

**Supplementary Data S4. Differential expression/network features between biopsies.** (**a**) Differential expression results are provided for the difference (delta) between the two biopsies. (**b**) Differential network feature results are provided per module, feature and gene for the difference between the two biopsies. Each comparison is the average difference in expression (or network feature values) between the two biopsies compared between PD vs SD/PR patients.

**Supplementary Data S5. Mapping between AMTEC and HTAN Identifiers.**

**Supplementary Data S6. Processed mIHC data.** Cell densities (cells/mm^2^) for CD20+ cells are provided for the 13 AMTEC samples where available.
